# Bizarre parosteal osteochondromatous proliferation in the distal ulna where the lesion is continuous with the medullary cavity: a case report

**DOI:** 10.1186/s12891-024-07715-4

**Published:** 2024-07-26

**Authors:** Tianyu Wang, Zhengxiao Ouyang, Zhuzhong Chen, Yuhui Yang, Xiaoyi Huang, Cheng Xiang, Lin Ling, Peng Zhou, Xiaoning Guo

**Affiliations:** 1 Department of Orthopedics, The Second Xiangya Hospital, Central South University, No 139 Renmin Road, Changsha, Hunan 410011 China; 2 Department of Orthopedics, The First People’s Hospital of Chenzhou, Chenzhou, Hunan China; 3 Department of Ultrasound, The People’s Hospital, Liaoning of Province, China Medical University, Shenyang, Liaoning China; 4 Central Sterile Supply Department, The Second Xiangya Hospital, Central South University, Changsha, Hunan China; 5 Department of Pathology, The Second Xiangya Hospital, Central South University, Changsha, Hunan China

**Keywords:** Bizarre parosteal osteochondromatous proliferation, Nora’s lesion, Continuity with the medullary cavity, Osteochondroma, Differential diagnosis

## Abstract

**Background:**

Bizarre parosteal osteochondromatous proliferation (BPOP) is a rare benign bone tumor, it is also called “Nora’s lesion”. The lesion is characterized by heterotopic ossification of the normal bone cortex or parosteal bone. The etiology of BPOP is unclear and may be related to trauma. In most BPOPs, the lesion is not connected to the medullary cavity. Here we report an atypical case, characterized by reversed features compared to the typical BPOP, which demonstrated continuity of the lesion with the cavity.

**Case presentation:**

An 11-year-old female child had a slow-growing mass on her right wrist for 8 months with forearm rotation dysfunction. Plain X-rays showed an irregular calcified mass on the right distal ulna, and computed tomography (CT) showed a pedunculated mass resembling a mushroom protruding into the soft tissue at the distal ulna. The medulla of this lesion is continuous with the medulla of the ulna. A surgical resection of the lesion, together with a portion of the ulnar bone cortex below the tumor was performed, and the final pathology confirmed BPOP. After the surgery, the child’s forearm rotation function improved significantly, and there was no sign of a recurrence at 1-year follow-up.

**Conclusion:**

It is scarce for BPOP lesions to communicate with the medullary cavity. However, under-recognition of these rare cases may result in misdiagnosis or inappropriate treatment thereby increasing the risk of recurrence. Therefore, special cases where BPOP lesions are continuous with the medulla are even more important to be studied to understand better and master these lesions. Although BPOP is a benign tumor with no evidence of malignant transformation, the recurrence rate of surgical resection is high. We considered the possibility of this particular disease prior to surgery and performed a surgical resection with adequate safety margins. Regular postoperative follow-up is of utmost importance, without a doubt.

## Background

Bizarre parosteal osteochondromatous proliferation (BPOP) is a rare benign extra periosteal proliferative lesion, which was first described in 1983 by Nora et al. and is therefore also known as “Nora’s lesion’‘ [1]. The disease is usually seen in the metacarpal and metatarsal bones of young patients [2]. The etiology of BPOP is unclear, with some scholars suggesting that the development of the disease is associated with traumatic stimulation [3]. The histologic features of this lesion consist mainly of bone, cartilage, fibrous connective tissue, and a special form of calcified chondroid. [2, 4]It is typically characterized by the presence of a dense ossified mass adjacent to the bone, which may be either not attached to the bone or may invade the bone cortex, both of which show a lack of continuity with the medullary cavity [2, 4–6]. This feature of discontinuity between lesions and medulla has been utilized as a distinguishing presentation for other tumors such as osteochondroma [1, 5]. However, Rybak and Berber et al. subsequently reported a limited number of BPOP cases in which the lesion communicated with the medullary cavity [7, 8]. Although such a lesion is extremely rare [4], it may be misdiagnosed as other tumors such as osteochondroma if the diagnosis criterion is based on discontinuity. It may be subject to confusion with other bone tumors. Although BPOP is a benign tumor with almost no further malignant changes [5, 6], the local recurrence rate ranges between 50–55% [3]. Incomplete surgical removal resulting from misdiagnosis may serve as a pivotal factor contributing to the recurrence of the condition [9]. We report a rare case of BPOP with a lesion communicating with the medullary cavity, located at the distal ulna.

## Case presentation

**Fig. 1:**
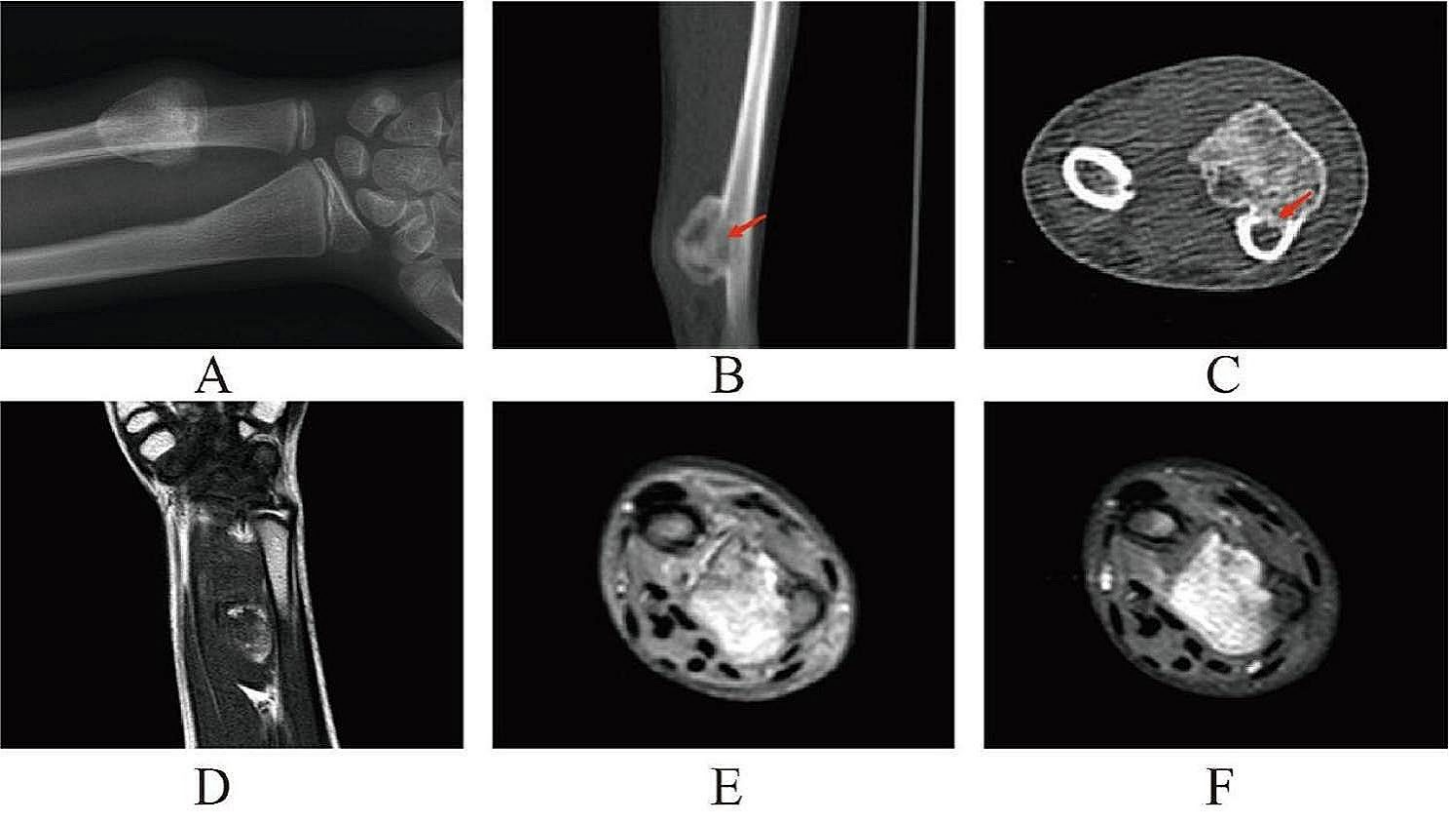
(**A**) A type of circular ossification was found in plain X-ray of the right ulna, which was closely related to the ulna. (**B, C**) The CT scan revealed that the lesion infiltrated the cortical bone of the ulna and communicated with the medullary cavity(arow). (**D**) On T1-weighted images showed inhomogeneous signal clumps adjacent to the ulnar and radius on the volar aspect of the right forearm. (**E**) On T2 with fat suppression, the lesion showed a predominantly high intensity signal. (**F**) On gadolinium-enhanced images showed inhomogeneous high signal throughout the tumor

A female patient11-year-old presented to a local clinic complaining of a slow-growing painless mass on her right wrist, beginning 8 months prior, with no apparent trigger, accompanied by partial restriction in the rotation function of her forearm. Initially, the orthopedic surgeon diagnosed osteochondroma based on clinical and imaging data and subsequently referred the patient to a specialist in bone oncology. The patient exhibited partial limitation in the rotational function of the right forearm, with anterior rotation ranging from 0° to 70° and posterior rotation ranging from 0° to 60°. No vascular or neurological symptoms were observed. Laboratory tests showed no significant abnormalities, and the patient denied any history of trauma or other diseases. X-rays showed a well-defined circular calcification shadow extending from the distal end of the right ulna to the palm side. The mass demonstrated a clear boundary and its base exhibited continuity with the bone cortex, without evidence of periosteal reaction (Fig. 1A). Computed tomography (CT) scan revealed an exophytic osseous mass situated at the distal aspect of the right ulna and adherent to the cortical bone, which has a wide base and connected to the medulla cavity (Fig. 1B, C). Magnetic resonance imaging (MRI) showed an approximately 25 mm×18 mm irregularly shaped abnormal mass, presenting long T1 and long T2 changes with inhomogeneous enhancement (Fig. 1D, E, F). The imaging revealed the involvement of the ulnar cortex, which communicated with the medullary cavity.

**Fig. 2:**
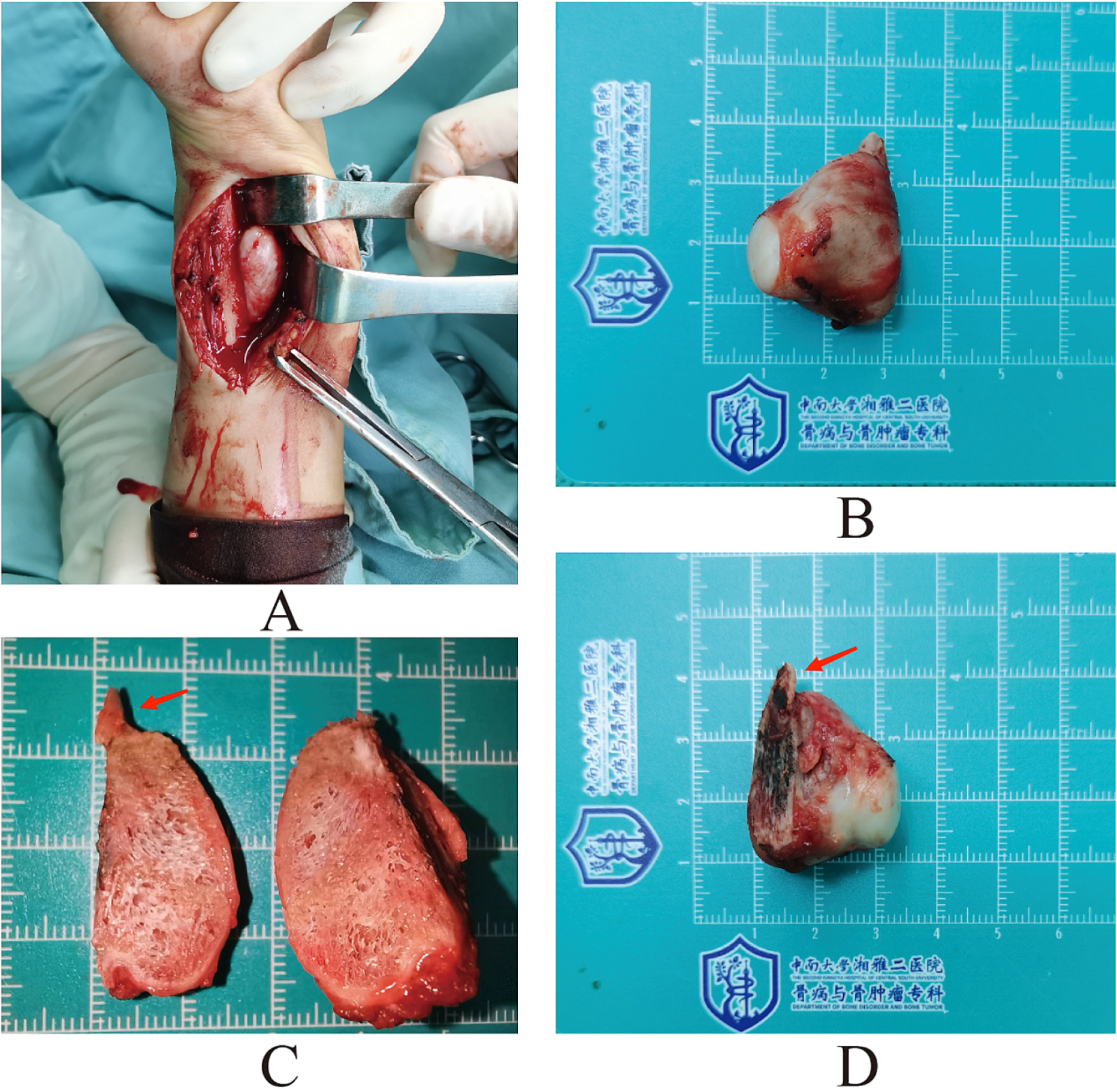
(**A**) The surgical exposure of the tumor revealed an exophytic, indurated mass located on the palm side of the distal ulna. Additionally, white chondroid components were observed covering the surface of the tumor, which was found to be interposed between the ulnar and radial bones, consequently impacting forearm rotation. (**B**) The tumor was completely excised from the ulna along with a portion of the cortical bone. The dimensions measured approximately 3.5 cm×2.6 cm. (**C, D**) A portion of the resected ulnar bone cortex is seen at the base of the tumor(arrow). At the junction of the tumor and the ulna, as well as inside the tumor, both are seen to be similar to a medullary cavity cancellous osteoid structure, with a continuous whole with no obvious interval separation between them

The patient underwent surgical treatment, during which the tumor was completely excised from the base along with a segment of the ulna cortex, and the resected osteotomy surface of the ulna was cauterized for inactivation. A smooth white cartilaginous cap is attached to the surface of the tumor. Dissection of the specimen showed that both the interior of the tumor and the interior of the osteotomy cortex were composed of structures similar to cancellous bone. (Fig. 2) The two form a continuous whole, without any apparent boundaries separating them. In summary, it can be confirmed that the lesion is continuous with the medullary cavity of the ulna.

**Fig. 3:**
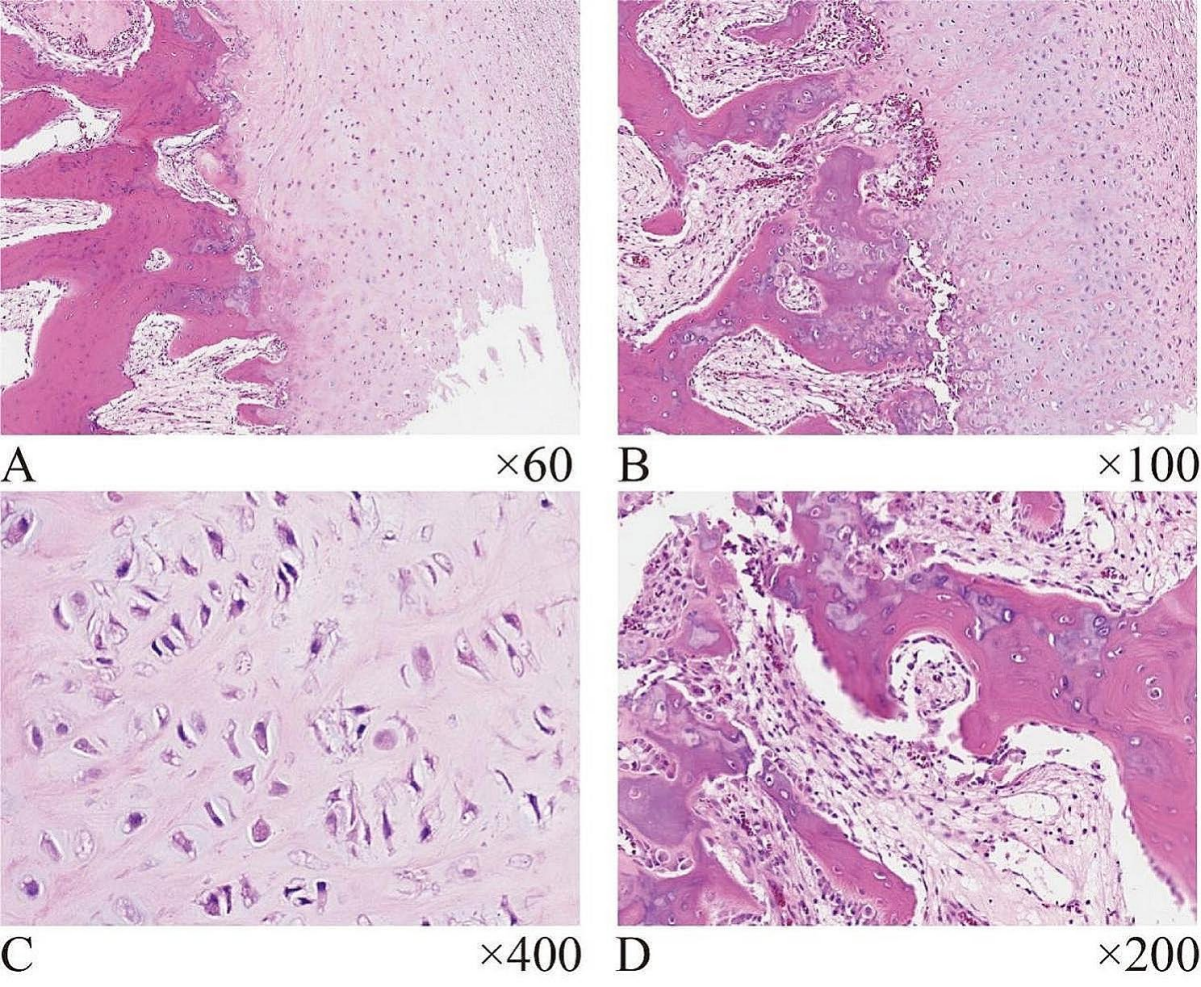
Hematoxylin and eosin (H&E) staining of the tumor sections. Histologically, a tumor is formed on the bone surface through mixed hyperplasia of fibers, cartilage, and bone in varying proportions (**A, B**). The surface of the lesion was a cartilage cap, with some areas of fibrous cartilage (**A**) and some areas showing hyaline cartilage (**B**). The chondrocytes exhibited an increased nucleoplasmic ratio and displayed aberrant morphology under high magnification (**C**). The cartilage undergoes migration and transformation towards the trabeculae through the form of endochondral ossification, resulting in the emergence of distinct blue bone areas within the transformed region (**A, B, D**). The bone trabeculae were interspersed with proliferating fibroblasts, without heterogeneity (**A, B, D**)

Histologically, it predominantly consisted of a substantial population of proliferating fibrous spindle cells, accompanied by a peripheral cartilage cap comprising a significant amount of fibrocartilage and some hyaline cartilage. There was an increased density of chondrocytes and mild mildly atypical features, however, neoplastic osteoid production was not observed. Furthermore, hematoxylin-eosin staining sections after tissue decalcification reveal the transition region between the two exhibited endochondral ossification and irregularity osteogenesis within the cartilage that a blue band of the cartilage-osteoid matrix, also known as blue bone, was demonstrated. The pathological results of the specimens confirmed that the lesion was the BPOP. (Fig. 3).

There were no obvious complications after surgical resection, and the wound healed well, and no signs of recurrence were seen in the regular follow-up of this patient after discharge.

## Discussion and conclusion

BPOP is a rare benign tumor of bone, and it is even rarer when the lesion is connected to the medulla. Due to the lack of standardized guidelines, there are several challenges associated with the diagnosis and treatment of lesions connected to the medullary cavity of BPOP. To our knowledge, about six cases of BPOP have been reported that communicate with the medullary cavity [4, 7, 8]. We report a rare case of BPOP in which the lesion was continuous with the medullary cavity, with a better therapeutic outcome through correct diagnosis and extended resection. The typical imaging findings of BPOP on X-ray and CT demonstrate irregular or lobulated shapes, accompanied by well-defined calcification or ossification. It commonly manifests in the short tubular bones of the hands and feet, exhibiting discontinuity with adjacent bones or involvement of the cortical region. T2-weighted images with fat suppression MR image shows the lesion to have slightly increased signal intensity centrally with the periphery being of much higher signal intensity. The higher peripheral signal intensity is probably related to cartilage content. On T1-weighted image the lesion demonstrates a low signal. Areas of calcification or ossification exhibit a diminished signal intensity. The lesion may show enhancement on The gadolinium-enhanced image [10]. The signal within the cortex and medullary cavity of the bone, where the lesion is attached is not abnormal. Additionally, the surrounding soft tissues exhibit a normal signal or demonstrate a mild edema signal. The BPOP may be disconnected from the bone or invade the adjacent bone cortex, but the lesion is mostly disconnected from the medullary cavity [1, 11].

BPOP needs to be differentiated not only from benign bone tumors such as osteochondroma and myositis ossificans but also from malignant tumors such as parosteal osteosarcoma. In addition, bone callus formation after fracture has some similarities with it, which deserves our attention [7]. The typical osteochondroma is commonly observed in the distal femur and proximal tibia, representing a bony neoplasm that grows in the opposite direction of a joint. On imaging, osteochondroma consists of cortical and medullary bone with an overlying hyaline cartilage cap and must be accompanied by continuity between the lesion and medullary cavity [12]. The X-ray and CT findings of the cases we reported do not align with typical BPOP but rather resemble the imaging characteristics observed in osteochondroma. However, it is not consistent with osteochondroma on MRI. A typical osteochondroma is characterized by a predominant signal of mature bone tissue on MRI, while the surface of the lesion exhibits long T1 and long T2 changes. The gadolinium-enhanced image did not demonstrate significant enhancement in peripheral lesions, indicating the presence of a cartilage cap adhered to the surface of the tumor [12]. And in the case we report, the whole tumor had significant inhomogeneous enhancement on MR. Although the lesion is connected to the medullary cavity, we tend to consider the disease as BPOP.

BPOP lesions internally show an aberrant trabecular bone structure and lack continuity with the medullary bone in most cases. Scholars usually distinguish osteochondroma from BPOP by this characteristic, however, there are still a few BPOPs that can demonstrate continuity with the medullary cavity. Rybak et al. first reported four cases of BPOPs in which the lesion communicated with the medullary cavity, and these four reports involved the metatarsal, radius, and ulna. In the report, the age range of these individuals was between 15 and 34 years old, with more females than males, and two of the patients relapsed sometime after surgery. Two more cases of BPOP with lesions connected with the medullary cavity of the phalanges have subsequently been reported [4, 8]. These uncommon BPOPs demonstrate imaging characteristics that are comparable to those observed in osteochondromas. There is a potential risk of misdiagnosis if the identification criteria strictly adhere to the previous features. Of course, myositis ossificans, florid reactive periostitis and parosteal sarcoma and so on need to be differentiated from BPOPs as well [13]. The occurrence of myositis ossificans is typically observed in the large, robust muscle groups of the limbs, commonly associated with a history of trauma and athletes. It initially manifests as localized redness, swelling, and pain. Myositis ossificans demonstrates a growth pattern that is distinct from numerous bone malignancies, as the calcification of the lesion progresses inwardly from its outer periphery. Florid reactive periostitis is common in the tubular bones of the hands and feet [14]. It often has a history of local infection or trauma, and its clinical manifestations include local redness, swelling, and pain. A few scholars have even suggested that it could potentially evolve into a BPOP [15]. Parosteal sarcoma is most common in the metaphyseal region of the long bones, manifesting as a lobulated or cauliflower-like lump with frequent irregular calcifications within the tumor [16]. The bone cortex in the area affected by the tumor may exhibit thickening or erosive changes, and the periosteal reaction is generally minimal [16]. 

Histologically, BPOP lesions are characterized by the presence of three main constituents: cartilage, bone, and fibrous tissue [6]. The arrangement of these structures may exhibit irregular patterns and exist endochondral osteogenesis [7]. The lesion margins are mostly cartilaginous cap components composed of fibrocartilage or hyaline, and the density of chondrocytes may increase, and there may be strange, enlarged, or binucleated chondrocyte populations, which may have mild atypia [7, 17]. This is one of the reasons why Nora’s lesion is labeled bizarre, but this atypia does not represent malignancy. The trabecular bone within the lesion does not represent true bone marrow but rather exhibits vascularization and proliferation of spindle cells without evident atypia. Additionally, the trabecular bone is enveloped by an outer cartilage cap displaying varying sizes and uneven calcification. The presence of a distinct type of calcified cartilage, known as “blue bone”, characterized by a blue coloration, can be observed under hematoxylin-eosin staining. This unique finding was initially reported by Meneses et al. [1]. Although BPOP is located adjacent to the bone and may show destruction of bone and bizarre proliferation of chondrocytes, there is no true neoplastic osteoid, so it can be distinguished from malignant tumors such as parosteal osteosarcoma. The histological layers of osteochondroma were more orderly, the cartilage cap was composed of hyaline cartilage, and the chondrocytes did not have atypia. Microscopically, the cartilage cap on the surface of the tumor was seen to be composed mainly of fibrocartilage and some hyaline cartilage, with mild atypical features such as hyperplasia and binucleation of some chondrocytes and neoplastic osteoid tissue formation was absent, in this patient. There were a large number of proliferating spindle cells in the trabecular bone without obvious atypia. The characteristic blue bone was observed in the tumor [6]. 

The primary approach for managing BPOP is typically surgical resection [18], with cryotherapy serving as an adjunctive treatment option [19]. Although the recurrence rate of BPOP following surgery is considerably high [3], there are limited reports on postoperative metastasis and malignant transformation [18]. It has been suggested that removing the pseudocapsule over the lesion, any periosteal tissue beneath the lesion, and decorticating any abnormal-appearing areas in the underlying host bone is an important measure to reduce recurrence [18]. We have taken into account the same factors. Therefore, we performed extensive surgical resection and local inactivation. The patient was followed up one year and five months and no signs of recurrence were detected. Additionally, the rotation function of the affected forearm showed significant improvement compared to preoperative levels.

In conclusion, although the diagnosis of BPOP can be obtained by imaging and pathology, it is important to be aware of the misdiagnosis of this type of BPOP that is connected to the medullary cavity. Acquiring an in-depth understanding of BPOP and its characteristics is imperative for accurate diagnosis establishment as well as the development of scientifically effective treatment strategies. A comprehensive understanding of the disease was needed to develop rational treatment. Further multi-center large-scale clinical trials and basic etiology exploration were needed to provide proper management and care services for patients.

## Data Availability

If someone wants to request the data from this study, please contact Corresponding author: Xiaoning Guo, The Second Xiangya Hospital, No 139 Renmin Road, Changsha, Hunan, 410011, China. E-mail: guoxiaoning@csu.edu.cn.

## Abbreviations

BPOP: Background: Bizarre parosteal osteochondromatous proliferation
CT: Computed tomography
MRI: Magnetic resonance imaging
